# Different Music Training Modulates Theta Brain Oscillations Associated with Executive Function

**DOI:** 10.3390/brainsci12101304

**Published:** 2022-09-27

**Authors:** Junce Wang, Ruijie Xu, Xiaolong Guo, Sijia Guo, Junchen Zhou, Jing Lu, Dezhong Yao

**Affiliations:** 1 The Clinical Hospital of Chengdu Brain Science Institute, University of Electronic Science and Technology of China, Chengdu 611731, China; 2 School of Life Science and Technology, University of Electronic Science and Technology of China, Chengdu 611731, China; 3 School of Glasgow, University of Electronic Science and Technology of China, Chengdu 611731, China; 4 Research Unit of NeuroInformation 2019RU035, Chinese Academy of Medical Sciences, Chengdu 611731, China; 5 School of Electrical Engineering, Zhengzhou University, Zhengzhou 450001, China

**Keywords:** music training, executive function, time-frequency analysis, functional connectivity

## Abstract

Different music training involves different hand coordination levels and may have a significant influence on brain oscillation for the executive function. However, few research has focused on the plasticity of executive function and the brain oscillation modulated by different musical instrument training modules. In this study, we recruited 18 string musicians, 20 pianists, and 19 non-musicians to perform a bimanual key pressing task during EEG recording. Behavioral results revealed that pianists have the highest accuracy and the shortest response time, followed by string musicians and non-musicians (*p* < 0.05). Time-frequency analyses of EEG revealed that pianists generated significantly greater theta power than the other groups from 500 ms to 800 ms post-stimulus in mid-central, frontal brain areas, and motor control areas. Functional connectivity analyses found that the pianists showed significantly greater connectivity in the frontal-parietal area in theta band based on phase-locking value analysis, which suggests that piano training improves executive function and enhances the connectivity between prefrontal and mid-central regions. These findings contribute to a better understanding of the effects of different music training on executive function.

## 1. Introduction

Music training is a multimodal training scheme related to memory, emotion, and multi-sensory feedback [1]. Since the Mozart effect was reported, music training has been an important model to reveal the training effect on the cognitive system as it recruits multimodal coordination of perception and motor [2]. Music training includes ear and vocal training, composing music, and playing the instruments, which have been regarded as practical tools to promote neural plasticity [3]. Research has found that musicians have larger gray matter volume in motor, auditory, and visual-spatial regions [4]. Recent studies also show that music training could enhance the neural encoding of the sound [5,6]. Specifically, studies have discovered the close relation between music training and executive function that even short-time music training could induce significant improvements in executive function [7,8]. Executive function is a top-down mental process of complex cognitive functions such as working memory, inhibitory control, attention, and cognitive flexibility [9,10]. The reason why music training and executive function are so related might be that music is a bottom-up and top-down art form requiring complex perceptual processing in which executive function plays a vital role. A previous study shows that after only one month of music training, the magnitude of P200, an event-related potentials (ERP) component related to the cross-modal integration, has increased along with improved executive functions [11]. In addition, frontal areas, which function as a pivot in the executive function networks related to higher order control cognition, are significantly more active in musicians than non-musicians when completing an N-back task [12,13]. A longitudinal study found significantly improved inhabitation control in preschoolers from 4 to 6 receiving music training six hours per week compared with control under the same amount of LEGO training [14]. Specifically, it is observed that children who take music courses have a better inhibitor control, in which the author postulates that music training has promoted children’s motor behavior via guiding the movements with the aid of music [15]. This explains that the reason why music training can significantly improve executive function might be that playing the instruments has a high requirement on the sensory-motor circuits [16].

Playing the instruments, as one of the main branches of music training, involves multiple arrays of perception that reflect motorial behavior, which composes the sensory-motor circuits [17]. It requires multiple capabilities, including reading the music score, making precise hand movements according to tempo, and adjusting based on listening [18]. Moreover, different instruments require diverse training regimes such as hand use. For example, the piano requires both hands to move under different tempi, but the guitar and violin do not [19,20,21]. They emphasize both hand coordination, which specifies right-hand plucking or bowing, and left-hand pressing the string simultaneously [22,23]. Research has shown that these diverse motor skills result in different neural alternations. It is also found that key-board players have a more prominent Omega sign (OS, an anatomical landmark of the precentral gyrus related with hand and finger movement) in the left hemisphere and string musicians in the right, which implies that lateral hand use might be a distinguished sign between different instruments leading to neural structural differences [24]. A previous study also found that the right hand of the string musicians has nearly no distinguishments from non-musicians by magnetic source imaging [25]. As research points out that executive function is also closely related to the training regime [26], the different requirements on hand movement have further influence on brain plasticity than just structural alternations on the motor area but some high order functions such as executive function. A previous study shows that rhythm-based music training has a significantly more prominent effect on inhibitory control than pitch-based music training in preschoolers [27]. Among all the music elements in instrument playing, hand-coordination is of great importance in the multi-sensory circuit as the above researches show in neural imaging of OS.

With above studies revealing the executive control instructing motor behavior in the motor-sensory circuits, we argue executive function is on the top level over the motor in music training. Simultaneously, there are only a few researches about the alternations of executive functions behind the hand movement in different musical instrument training modules. In this study, we explore the relationship between the executive functions and motorial techniques in music training. As different instruments like piano and string instruments specify different hand coordination levels, we hypothesize that different instruments may have a significant influence on the brain oscillation for the executive function on top of the motorial diversities. To reveal the relationship between the executive function and the brain oscillation modulated by different instrument training, we recruited pianists, string musicians, and non-musicians and utilize EEG to reveal the deviations in brain oscillations when performing the bimanual key pressing (BKP) tasks.

## 2. Method and Materials

### 2.1. Participants

We recruited 38 musicians, including 20 pianists (Piano) and 18 string musicians (String) for the experimental groups. Meanwhile, we recruited 19 healthy adults as the control group (Control). All the participants completed a musical experience questionnaire to determine the age of onset of musical training, length of formally musical training, and hours of current practice. All the musicians were guaranteed to begin playing before 10 and have formal musical education of more than 5 years. They reported still actively studying music in recent five years, including practicing daily or taking systematic music lessons. Similar criteria were used in a study comparing musicians and non-musicians [28]. Musicians who had learned both string instrument and piano were excluded. The music groups, Piano and String, only vary in the types of training instruments. Pianists and String Musicians did not differ in age (*p* = 0.52; String: 21.76 ± 4.92; Piano: 20.75 ± 2.45), years of formal musical education (*p* = 0.61; String: 12.05 ± 4.92; Piano: 11.23 ± 4.55), current practice hours per week (*p* = 0.82; String Musicians: 6.25 ± 7.21; Pianists: 3.95 ± 4.67). The control group was formed with people with no musical experience, and similar ages and educational levels with the experimental groups (*p* > 0.05). All participants were healthy with no brain disease and taking no medication for the recent three months. Moreover, all of them were right-handed as assessed by the Edinburgh handedness inventory [29]. The psychometric tests comprised the following ones: (i) Basic Information; (ii) Self-rating Anxiety Scale (SAS) [30]; (iii) Self-rating Depression Scale (SDS) [31]; (iv) The Big Five [32]; (v) Edinburgh Handedness Inventory [29]; (vi) Barcelona Music Reward Questionnaire (BMRQ) [33]; (vii) Montreal Battery of Evaluation of Musical Abilities (MBEMA) [34] (see Table 1 for participant demographic details). The reason why we applied these psychometric tests was included in the supplementary material. The experimental protocol was approved by the Ethics Committee of the University of Electronic Technology and Science of China (Ethics approval code: No.1061420210305026 Date: 25 February 2021), and participants provided written informed consent before participation.

Supplementary material for the psychometric tests:

1. Basic Information Tests: This test is mainly aimed at gathering basic information about the subjects such as gender, age, occupation, and education level. It includes 10 questions. Among them, the age and education level have been analyzed to guarantee the subject data standardization, of which the specific *p*-value, mean value, and standard deviation are listed in the article.
2. Self-rating Anxiety Scale (SAS) & Self-rating Depression Scale (SDS): The SAS was developed by Zung in 1971 to measure anxiety and the SDS was developed by Zung in 1965 to measure depression. Both of them have proven sufficient reliability and have constructed validity to justify further application in scientific research. In our article, we have to use these tests to assure that all the subjects were in a mentally healthy status. Both of them include 20 questions. The results for different groups show no discrepancy.
3. The Big five: This test is meant to measure the personality of each subject. It includes 48 questions and defines one personality as Neuroticism (whether susceptible to pressure), Extraversion (whether outgoing and optimistic), Openness (whether creative and innovative), Agreeableness (whether cooperative and friendly), Conscientiousness (whether responsible and magnanimous). The results show no discrepancy in different groups, implying that they have similar backgrounds and life experiences. In our study, this study could have further use in future research.
4. Edinburgh Handedness Inventory: In our article, we used the short form Edinburgh Handedness Inventory which was developed in 2014 by Veale. This is an improved version based on confirmatory factor analysis. Furthermore, we utilized this test to make sure that all the subjects were right-handed.
5. Barcelona Music Reward Questionnaire (BMRQ): This questionnaire is intended to gather the music preferences of the subjects. It includes 20 questions and defines four types of music preferences: Sensory Motor (inclined to listen to the music along with humming, clapping, or dancing), Mood Regulation (inclined to get emotional, sentimental, or affectionate when listening to music), Musical Seeking (inclined to constantly seek for new music), and Social Reward (inclined to build connections with others through playing, listening or talking about music). This scale could be applied in future research.
6. Montreal Battery of Evaluation of Musical Abilities (MBEMA): This questionnaire is intended to gather the musical abilities of the subjects. It includes 59 questions. It includes questions about musical abilities such as absolute pitch and relative pitch, music theory, composing, and improvisation. It also includes questions about musical information such as the onset age, the years of formal musical training and different stages of learning music, and how many hours of practice per day and week. The results for String and Piano show no discrepancy either.

### 2.2. Procedure

Testing (EEG and psychometric test) took place in the laboratory consecutively and lasted 1.5 h in total.

**Figure 1:**
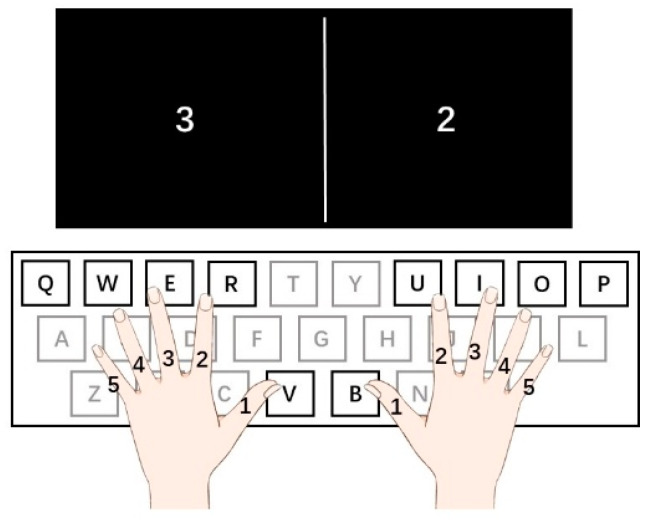
Experimental protocol. During the experiment, participants placed their fingers on the buttons for the left hand: thumb- button V, index finger-button R, middle finger-button E, ring finger-button W, little finger -button Q, and for the right hand: thumb- button B, index finger-button U, middle finger-button I, ring finger-button O, little finger-button P. Two pseudo-random numbers are presented at the two centers of the left and right half screen. The numbers specify each figure for both hands: 1-thumb, 2-index finger, 3-middle finger, 4-ring finger, 5-little finger. For the numbers shown on the left side, they are required to use the left-hand finger specified by the number to press the corresponding buttons, and for numbers shown on the right side, use the right-hand finger.

We measured the neuroelectric activity by EEG while participants were presented with the BKP task. Before each trial, a white fixation cross on a black background appeared on the center of a screen located approximately 30 cm from the participant for a variable duration (800–1000 ms). The keyboard was displaced horizontally and subtle changes in the position of the keyboard were allowed due to comfortableness. Then, the participants were presented with two pseudorandom numbers from 1 to 5 on the centers of each half of the screen and were required to press the specific buttons with corresponding fingers (Figure 1). Once participants pressed two buttons in total, regardless of whether correct or incorrect in 1500 ms, the trial ended and entered into the subsequent trial. No execution in 1500 ms would be regarded as an incorrect response. The experiment consisted of 100 trials in total. Before the experiment, each participant would have five minutes to accustom to the tasks. During the experiment, participants were not given any feedback on their performance. Accuracy rates and response time were recorded during the task. The stimuli were presented with E-prime 3.0 (Psychology Software Tools, Pittsburgh, PA, USA, https://pstnet.com/products/e-prime/ (accessed on 31 July 2021)).

### 2.3. EEG Recording and Data Preprocessing

EEG was recorded using an active system (ActiCap, Brain Products, Gilching, Germany). The participants wore an EEG cap with 63 Ag/AgCl electrodes placed according to the International 10–20 system, and data was sampled at 1000 Hz and bandpass filtered from 0.3 to 100 Hz. The electrodes ‘FCz’ was used as a reference electrode. The electrode impedances were kept below 10 kΩ. The neuroelectric activity is then stored for offline analysis.

Offline processing of the EEG data was performed in MATLAB (R2016a; Mathworks, Natick, MA, USA) using the EEGLab toolbox (https://sccn.ucsd.edu/eeglab/index.php (accessed on 10 February 2022)) [35] and Brainstorm software (https://neuroimage.usc.edu/brainstorm/ (accessed on 10 February 2022)) [36]. Then, the data were transformed to zero reference using the EEGLAB toolbox REST [37]. Continuous EEG data were high-pass filtered above 1 Hz and low-pass filtered under 50 Hz. Subsequently, EEG data were segmented into short epochs from −200 ms before and 800 ms after the onset of the stimuli with baseline correction using the pre-stimulus interval. As the background EEG signals were nonlinear, nonstationary and larger than the ERP signals, time-locked averaging was conducted. Then, the independent component analysis was applied to identify and remove eyeblinks and movements. After ocular correction, traces were scanned for artifacts and epochs with deflections exceeding ±60 µV marked and excluded.

### 2.4. Time-Frequency Analysis

Time-frequency analysis was implemented to identify brain oscillations in performing bimanual pressing tasks. As this study focused on executive function, we chose some typical nodes in frontoparietal networks including F1, F2, FC1, FC2, Cz, C1, and C2, which were shown to be related to the central executive process in neuroimaging study [38,39,40]. As executive function dominates motorial behavior which is referred to as sensory-motor control, we chose electrodes in the motor area as the ROI to reveal the motorial representation behind different instruments. Therefore, C3 and C4 were chosen to monitor motor control as the former study indicated that these two sites were in the vicinity of the primary motor cortex [41,42]. After that, we focused on theta band oscillations (4–7 Hz) as it was shown to relate to executive control [43,44,45]. Additionally, as the behavioral results showed that the lowest mean response time was approximately 800 ms, we chose 500–800 ms as the time interval to explore the prior motor neural oscillations. Specifically, a time-frequency distribution of the EEG time course was obtained using a windowed Fourier transform (WFT) with a fixed 300 ms Hanning window. During this process, it was assumed that the EEG signal was stationary only in a short interval. For each time course, the WFT yielded a complex time-frequency estimate F(t,f) at each point (t,f) of the time-frequency plane, extending from −200 ms to 800 ms (in 1 ms intervals) and from 2 to 50 Hz (in 1 Hz interval). The resulting P(t,f) = |F(t,f)|^2^ was then applied to represent the signal power at each time-frequency point by the joint function of time and frequency. This yielded the time-frequency distribution (TFD). Point-by-point one-way analysis of variance (ANOVA) was performed between the groups of pianists, string musicians, and the control to reveal whether there is a significant difference in power distribution.

### 2.5. Functional Connectivity

**Figure 2:**
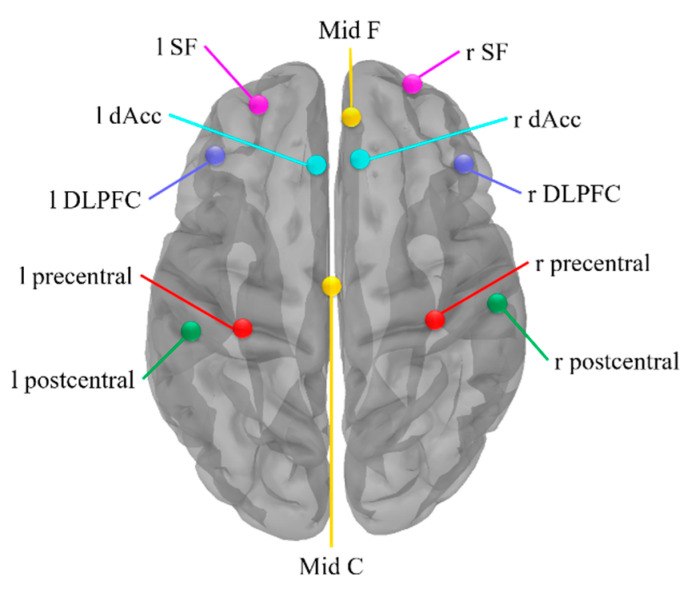
Schematic of the source montage used in calculating PLV. Abbreviations: Mid F, mid frontal cortex; r SF, right superior frontal; l SF, left superior frontal; r dAcc, right dorsal anterior cingulate cortex; l dAcc, left dorsal anterior cingulate cortex; r DLPFC, right dorsal lateral prefrontal cortex; l DLPFC, left dorsal lateral prefrontal cortex; Mid C, mid-central cortex; l precentral, left precentral cortex; l postcentral, left postcentral cortex; r precentral, right precentral cortex; r postcentral, right postcentral cortex.

Firstly, the analysis of intracranial sources was based on the standardized low-resolution brain electromagnetic tomography source analysis (s-LORETA) analysis on the Brainstorm software. The s-LORETA was a standardized, discrete and 3D distributed, linear and minimum norm solution with no localization bias even in the presence of biological or measurement noise [46]. To study the cooperative and synchronous neuronal assemblies, we used phase-locking value (PLV) to extract the non-linear phase synchronization between paired nodes (Figure 2). The dorsal lateral prefrontal cortex (DLPFC), superior frontal (SF), anterior cingulate cortex (ACC) and mid frontal area are found activated by a wide range of executive function tasks [47,48,49,50]. The nodes in these regions were chosen in pursuit of revealing the connectivity within the Executive Function Network (EFN), which is related to detecting stimulus and guiding behavior accordingly. The motor area including the mid central area, precentral and postcentral gyrus, and inferior frontal cortex, which were shown as task-relevant areas for music training during motor-related tasks, were chosen additionally [42,51,52,53]. The s-LORETA calculated a single vortex at each ROI centroid. Therefore, PLV was calculated between nodes within the EFN and the motor area, and the calculation was implemented by the Brainstorm software. We measured functional connectivity within these nodes based on PLV and then subtracted between groups to get three different matrices. Paired t-test was performed on the different matrices of String vs. Control, Piano vs. Control, and Piano vs. String.

### 2.6. Statistics

ANOVA tests were performed to assess the significance of the group information among String, Piano, and Control. Bonferroni correction was utilized to implement the multiple comparisons. ANOVA tests were also utilized to compare behavioral results including accuracy and response time, among those three groups. In the case of normal distribution, paired *t*-test was used in the power spectrum and PLV analysis.

## 3. Results

### 3.1. Response Time and Accuracy

**Figure 3:**
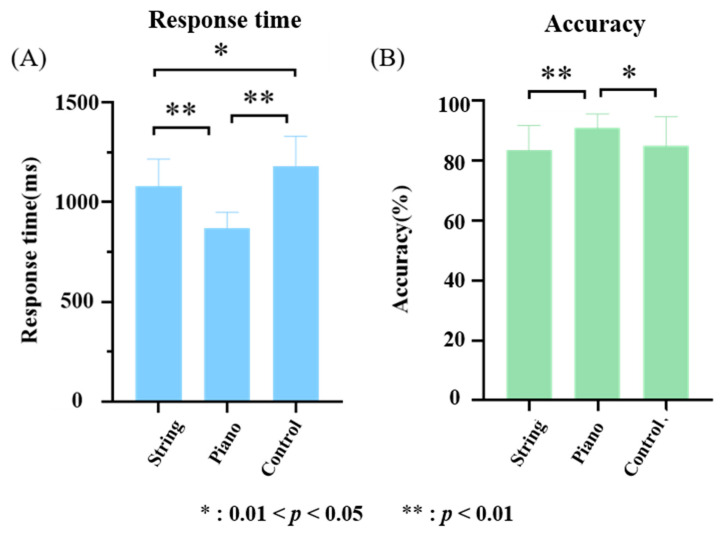
Response time and accuracy (**A**) Differences in response time between string musicians (showed as String in the figure), pianists (showed as Piano in the figure), and the control group (showed as Control in the figure). (**B**) Differences of accuracy between string musicians (showed as String in the figure), pianists (showed as Piano in the figure), and the control group (showed as Control in the figure).

One-way ANOVA was used to analyze the differences in response time and accuracy among the string musicians, pianists, and control groups in the BKP task, and Bonferroni’s posttest was used to make multiple comparisons among three groups (Figure 3). The results showed that the response time of pianists was significantly lower than that of string musicians (*t* = −5.9099, *p* < 0.001) and the control group (*t* = −8.2294, *p* < 0.001), and the response time of string musicians was significantly lower than that of the control group (*t* = −2.1366, *p* = 0.0397). The accuracy of pianists was significantly higher than that of string musicians (*t* = 3.3746, *p* = 0.0018) and the control group (*t* = 2.4577, *p* = 0.0187).

### 3.2. Effects of Different Music Training on Theta Power

**Figure 4:**
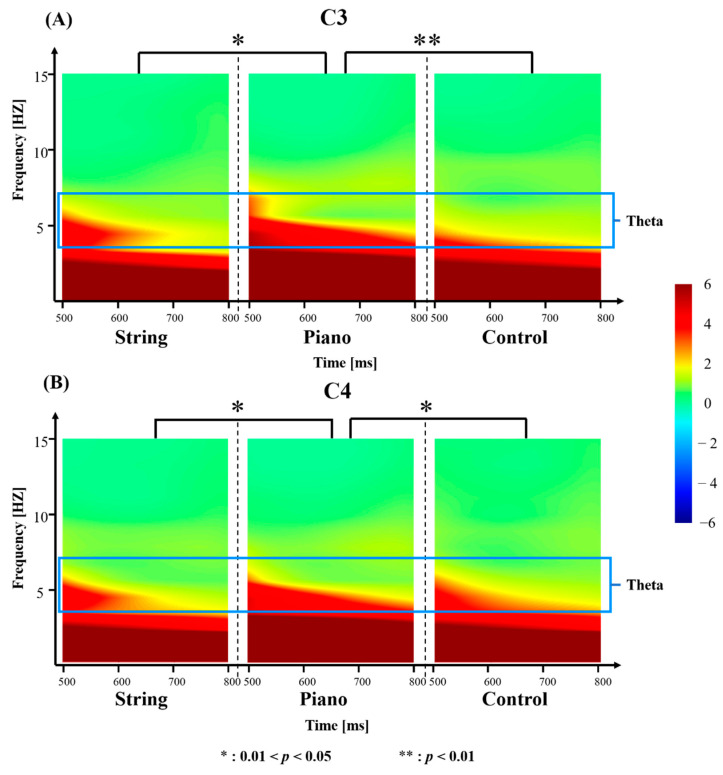
(**A**) Group mean difference between three groups (string musicians showed as String in the figure, pianists showed as Piano in the figure, the control group showed as Control in the figure) in theta power. The measurements reflect the mean theta (4–7 Hz) power (as shown in the blue frame in the figure) between 500 and 800 post-stimulus from C3. (**B**) Group mean difference in theta power between three groups (string musicians showed as String in the figure, pianists showed as Piano in the figure, the control group showed as Control in the figure). The measurements reflect the mean theta (4–7 Hz) power (as shown in the blue frame in the figure) between 500 and 800 post-stimulus from C4.

One-way ANOVA was also used to analyze the group mean differences in theta power between 500 ms and 800 ms post-stimulus from C3 and C4. We found that pianists have a greater theta power than that of string musicians (*t_C_*_3_ = 2.4565, *p_C_*_3_ = 0.0183; *t_C_*_4_ = 2.4175, *p_C_*_4_ = 0.0214) and the control group (*t_C_*_3_ = 2.8854, *p_C_*_3_ = 0.0036; *t_C_*_4_ = 2.5072, *p_C_*_4_ = 0.0147) between 500 ms and 800 ms post-stimulus from C3 and C4 (Figure 4).

**Figure 5:**
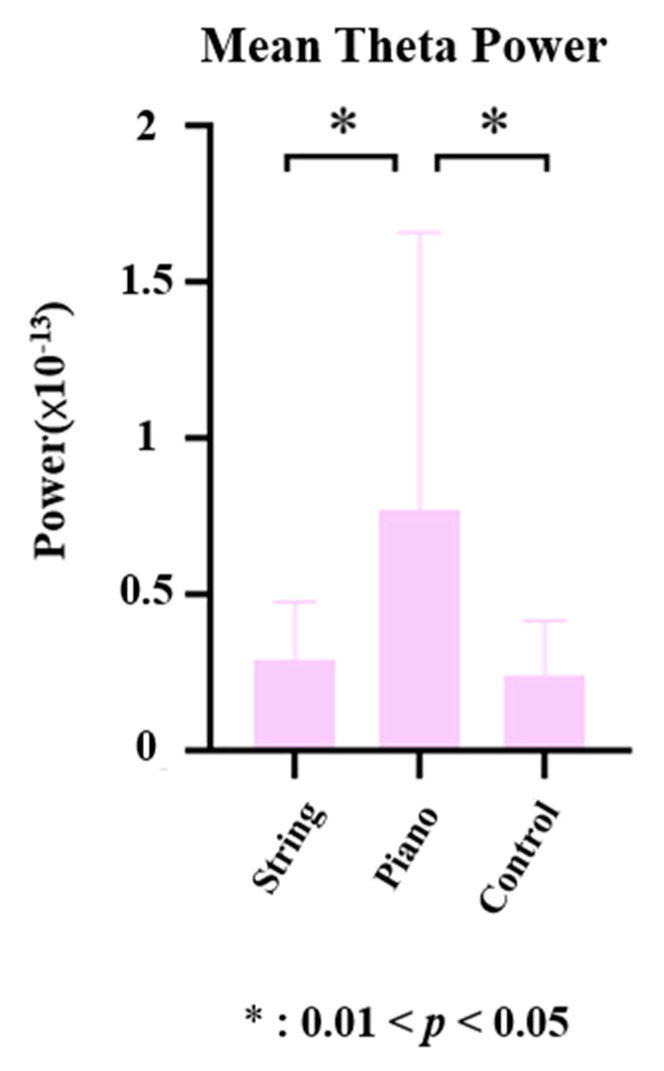
Group mean difference in theta power between three groups (string musicians showed as String in the figure, pianists showed as Piano in the figure, the control group showed as Control in the figure). The measurements reflect the mean theta (4–7 Hz) power between 500 and 800 post-stimulus from midline region.

To better understand the effects of music training on executive function, we compared the mean theta power from the midline central region. The analysis revealed that pianists have a greater mean theta power than that string musicians (*t* = 2.2478, *p* = 0.0308) and the control group (*t* = 2.5711, *p* = 0.0143) between 500 ms and 800 ms post-stimulus from midline central region (Figure 5).

### 3.3. Functional Connectivity

**Figure 6:**
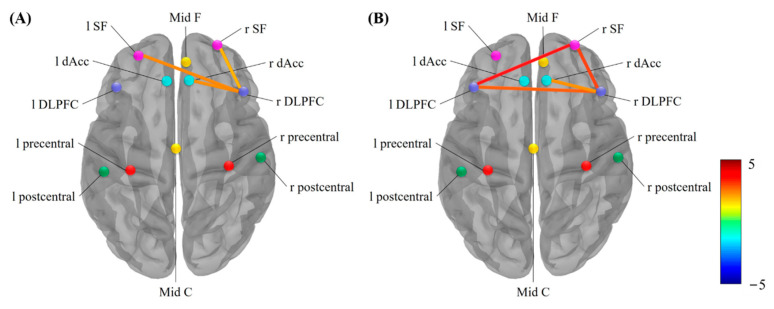
Functional connectivity within the executive functional network based on PLV, the color of the color bar represents the size of the t value. Abbreviations: Mid F, mid frontal cortex; r SF, right superior frontal; l SF, left superior frontal; r dAcc, right dorsal anterior cingulate cortex; l dAcc, left dorsal anterior cingulate cortex; r DLPFC, right dorsal lateral prefrontal cortex; l DLPFC, left dorsal lateral prefrontal cortex; Mid C, mid-central cortex; l precentral, left precentral cortex; l postcentral, left postcentral cortex; r precentral, right precentral cortex; r postcentral, right postcentral cortex. (**A**) Differences between string musicians and the control group (*p* < 0.05). (**B**) Differences between pianists and the control group (*p* < 0.05).

We tested whether the impact of different music training on hand motor executive function was linked to changes in functional connectivity within the executive control network by comparing PLV elicited by the BKP task. Differences in theta power between string musicians and control group were observed in three connectivities: lSF-rDLPFC (*t* = 2.5025, *p* = 0.0342), rSF-rDLPFC (*t* = 2.1400, *p* = 0.0290) and rdAcc-rDLPFC (*t* = 2.2764, *p* = 0.0292) (Figure 6A). Differences in theta power between pianists and control group were observed in four connections: lDLPFC-rSF (*t* = 3.8701, *p* = 0.0014), lDLPFC-rDLPFC (*t* = 2.9373, *p* = 0.0066), rdAcc-rDLPFC (*t* = 2.1425, *p* = 0.0415), rSF-rDLPFC (*t* = 3.2964, *p* = 0.0014) (Figure 6B).

## 4. Discussion

The study aimed to investigate the plasticity of executive function and the brain oscillation modulated by different musical instrument training modules. Pianists and string musicians are asked to complete a bimanual key pressing (BKP) task during EEG recording. Two aspects of their performance were assessed: accuracy and response time. The results showed that pianists have the highest accuracy and the shortest response time. The enhancement of theta power and brain functional connectivity in the frontal lobe during the BKP task may reflect the training effect of executive function under music training.

Performing music can be one of the most complex and demanding forms of fine motor expression [18,54]. Musicians must translate music notation (visual-spatial-temporal information) into precisely timed sequential finger movements based on existing musical pieces [55]. However, depending on the instrument being played, the motor behavior of the hands may differ. For example, when playing a stringed instrument, one of the musician’s arms moves horizontally while the other arm moves vertically, but the pianists use both arms horizontally when playing the piano [20]. Previous studies have found that in string instruments training, such as violin training, in the left forearm, the wrist and finger flexor and extensor muscles are used to control the fingering movements in the hand; while in the right forearm, the flexors and extensors are used to control the bow [22,23]. Therefore, the left hand of the string musicians makes fine movements, while the right hand makes gross movements during instrument performing or practicing; However, piano training involves moderate to relatively high levels of muscle activation of the flexor and extensor muscles of both hands, wrists, and fingers [21,56,57]. Therefore, the pianist’s hands both do fine movements during instrument performing or practicing.

A previous study reported that fine motor training (piano training) could significantly improve the fine motor control ability compared with gross motor training (percussion training) and music listening [58]. Similar to their results, the results of accuracy and response time in this work showed that pianists have better fine bimanual motor control ability than string musicians and the control group, the underlying reason may be related to the effect of executive function for different instruments training modules.

Playing a musical instrument requires many sub-skills associated with executive function, such as sustained attention, goal-directed behavior, and the task-switching demands of cognitive flexibility [59]. During the BKP task, the subjects need to accurately control the fingers of both hands according to the visual prompts, which is very similar to the process of musicians playing music, this involves that the subjects keep continuous attention to the visual cues and select the correct finger through the visual cues to press the corresponding key as soon as possible.

In the results of the time-frequency analysis, we observed a significant increase in theta power elicited in motor areas before movement execution in all three groups. Previous studies have shown that motor cortical theta oscillations have been closely associated with theta activity in the medial frontal cortex in both the visual and auditory modalities, and medial frontal theta activity may represent communication between the frontal midline and other brain areas during cognitive control, this is consistent with the result that the pianists showed stronger theta power in medial frontal and motor regions compared with the string musicians and the control group.

Previous studies have shown that theta oscillation in the medial frontal region is closely related to executive control function [60,61]. Wherein attentional demands are related to increases in frontal-midline theta power [62,63,64], enhanced theta band connectivity between the default network (DN) and frontoparietal control network (FPCN) is a core electrophysiological mechanism that underlies internally directed attention [65]. In a previous study, a similar task of finger key presses to the BKP task was used, and it was found that the stronger frontal middle theta band reflected the subjects’ sustained attention [66]. In this study, pianists’ stronger theta band in the medial frontal area may also represent their stronger sustained attention in executive function. Previous studies have shown that short-term or long-term music training can enhance executive control function in children [59,67,68,69], adults [7,59,70,71], and the old [58,72,73], but the specific effects of different types of musical training on executive function are still unclear [74].

It has been proved in studies of animals and humans that the prefrontal cortex is a crucial structure for performing executive functions [39,75,76,77]. The lateral prefrontal cortex, including the DLPFC, is a well-documented region actively involved in tasks requiring executive control [78]. Previous studies have found that functional connectivity of the DLPFC with other brain regions contributes to different executive function components [79,80,81,82,83]. In the meantime, the involvement of the ACC in executive functions is more precisely linked to the dorsal ACC (dAcc). This region is a critical hub in a network of brain regions involved in human domain-general executive functions [84,85]. Meanwhile, theta activity of ACC has been proven related to focused attention and high cognitive demand [66,86,87].

Our study found that pianists have stronger functional connectivities among DLPFC, dACC, and SF, which may represent their stronger attention and executive functions compared to string musicians and controls. However, we found that compared with the control group, the functional connectivity between DLPFC and SF of string musicians also showed significant enhancement, which indicates that the training mode of string instruments can also effectively improve executive function, but the effect is not as significant as that of bimanual coordination fine training mode. Therefore, although previous studies have found that music training can effectively improve executive function [58,59,60,61,62,63,64,65,66,67,68,69,70,71,72,73], our research suggest the different effect of different musical training on executive function. This finding may be applied to music therapy to help patients improve their executive function more effectively.

## 5. Conclusions

Our work found that theta oscillatory activity in the frontal and motor region dominated the executive function of all participants during the BKP task. Increased activity in frontal regions was observed in the pianists compared to the string musicians and the control group. Our findings are consistent with previous research, indicating that theta oscillation may be a marker for the difference in executive function caused by different music training. Results of PLVs connectivity revealed the same trend, that is, pianists showed a significant enhancement to string musicians and the control group. These functional connectivities are mainly concentrated in the prefrontal areas, which may reflect the critical role of prefrontal areas in the music-training-regulated executive function and reveals the underlying brain mechanisms of this process. In general, our study suggests that the bimanual coordination fine training mode (like piano training) in music training could improve the executive function, this may be helpful for understanding the effect of music training and also contribute to the practice of music therapy.

## 6. Limitations

A limitation of the current study is that it is difficult to find a suitable quantitative index of hand movement in different music training to describe the exercise of hands, which may be related to the training effect of executive function. Future work could focus on this issue and recruit people who may have better fine motor skills (such as surgeons or video game players) to verify the relationship between fine motor training and executive control functions. Besides, future research can carry out experiments on participants with different diseases (such as Parkinson’s or Alzheimer’s disease) to provide more references for music therapy.

## Figures and Tables

**Table 1:** Participant Demographics. The control group was formed with people with no musical experience, and similar ages and educational levels with the experimental groups (*p* > 0.05). Musicians are guaranteed to conduct music training continuously in recent five years. The string musicians and pianists do not significantly differ in these three indexes (*p* > 0.05). The scores of the Self-rating Anxiety Scale (SAS), Self-rating Depression Scale (SDS), Edinburgh Handedness Inventory, The Big Five, and Barcelona Music Reward Questionnaire (BMRQ) all show no significant difference among three groups.

	String	Piano	Control
*n*	18	20	19
Male/Female	11/7	11/9	11/8
Age (years)			
	21.76 ± 4.92	20.75 ± 2.45	20.68 ± 1.34
Education Level (years)			
	14.12 ± 1.20	14.35 ± 2.30	14.5 ± 1.31
Age of Musical Training Onset (years)			
	7.38 ± 3.36	5.7 ± 1.87	-
Formal Training (years)			
	12.05 ± 4.92	11.23 ± 4.55	-
Self-rating Anxiety Scale (SAS)			
	29.53 ± 7.83	27.11 ± 3.78	31.15 ± 6.65
Self-rating Depression (SDS)			
	30.69 ± 7.08	30.5 ± 5.68	32.53 ± 6.83
Edinburgh Handedness Inventory			
	10	10	10
The Big Five			
The Big Five-Neuroticism			
	30.27 ± 4.93	30.70 ± 5.85	31.22 ± 4.93
The Big Five-Extraversion			
	27.27 ± 6.52	25.75 ± 6.51	25.89 ± 4.23
The Big Five-Openness			
	43.80 ± 4.98	42 ± 3.50	38.56 ± 3.50
The Big Five-Agreeableness			
	33.27 ± 5.50	35.55 ± 4.24	33.83 ± 3.73
The Big Five-Conscientiousness			
	35.13 ± 5.08	32.90 ± 4.75	33.78 ± 4.51
Barcelona Music Reward Questionnaire (BMRQ)			
BMRQ-Emotional Evocation			
	18.47 ± 1.77	17.15 ± 2.21	15.89 ± 2.14
BMRQ-Sensory Motor			
	16.73 ± 2.02	15.95 ± 3.19	13.39 ± 3.11
BMRQ-Mood Regulation			
	17.87 ± 1.55	17.10 ± 2.10	15.56 ± 1.89
BMRQ-Musical Seeking			
	17.67 ± 1.72	16.80 ± 1.90	14.56 ± 2.38
BMRQ-Social Reward			
	17.27 ± 1.22	16.55 ± 2.61	14.16 ± 2.62

## Data Availability

The data presented in this study are available on request from the corresponding author.
